# Case Report: The Versatile Art of Reconstruction: A Decade‐Long Journey With Recurrent Facial Basal Cell Carcinoma

**DOI:** 10.1002/cnr2.70124

**Published:** 2025-01-29

**Authors:** T. H. Pathirana, V. Bandaranayake, A. P. Nellihela, S. Nikeshala, T. Saranga, A. Abegunasekara, S. Gunathilake, N. J. Asanthi

**Affiliations:** ^1^ Surgical Oncology Unit Teaching Hospital Anuradhapura Anuradhapura Sri Lanka; ^2^ Histopathology Department Teaching Hospital Anuradhapura Anuradhapura Sri Lanka

**Keywords:** basal cell cancer, cancer care, dual flap, facial reconstruction, head and neck cancer, karapandzic flap, lip reconstruction, pedicled flaps for lip reconstruction, surgical oncology

## Abstract

**Background:**

Basocellular carcinoma (BCC) is the most prevalent skin malignancy, often localizing to the UV‐exposed skin of the face. While most BCC is relatively indolent, aggressive subtypes, including infiltrative BCC, pose the treatment challenges of ensuring functional and aesthetic preservation with a high risk of recurrence.

**Case:**

A 78‐year‐old female patient complained of recurrent left chin BCC of infiltrative subtype, which was first treated in 2013 by wide local excision and adjuvant radiotherapy. Recurrent carcinoma was reported in 2017, 2021, and most recently in 2023. The most recent recurrence presented with scar contracture, facial asymmetry, and drooping of the lip. Surgical resection involved extensive excision of the lower lip and chin, exposing the mandible. Reconstruction was achieved with the help of bilaterally designed Karapandzic flaps for the lip and a bilobed rotational flap for the chin defect. Although no chemotherapy and immunotherapy were given, the functional and aesthetic result were satisfactory. The procedure was executed while preserving the pertinent neurovascular structures, maintaining oral competence, and restoring the lower face. The Karapandzic flap was helpful in maintaining the lip function, whereas the chin was reconstructed using the bilobed rotational flap. Post‐operative histology confirmed the diagnosis of BCC with squamous differentiation, which had clear margins. The patient did well and the cosmetic and functional outcome was satisfactory without any disease at 18 months after surgery.

**Conclusion:**

This case sheds light on the difficulties encountered in treating recurrent facial BCC in developing countries. In the absence of systemic therapies, advanced surgical techniques and intensive follow‐up resulted in a long disease‐free period with good functional and aesthetic outcomes, thanks to the successful use of innovative reconstruction even in a low‐resource setting.

## 1 Introduction

Basal cell carcinoma (BCC), the most prevalent form of skin cancer, accounts for nearly 80% of non‐melanoma skin cancers. It occurs in areas exposed to ultraviolet (UV) radiation, such as the face, hence making facial BCCs a common clinical challenge [1, 2]. Even though BCCs are generally considered to be slow‐growing tumors with low metastatic potential, certain histological subtypes, such as infiltrative and micronodular BCCs, demonstrate more aggressive behavior, particularly in regards to recurrence and local invasion. Advancements in diagnostic tools, including dermoscopy and optical coherence tomography, have improved early detection of BCC, aiding in more precise surgical interventions [3].

The accepted treatment for BCC is surgical excision with histologically clear margins, which is important for minimizing the risk of recurrence. The [4] recommend a 4–5 mm margin for low‐risk BCCs and a minimum of 6 mm margin for high‐risk BCCs [5]. However, recurrent BCCs, especially those on the face, pose a significant challenge to the surgeon due to the difficulty of achieving adequate oncological clearance due to adverse cosmetic and functional outcomes.

The field of reconstructive surgery has emphasized the importance of preserving facial function and aesthetics, with options like the Karapandzic flap being particularly effective for extensive lower facial resections [6, 7].

This is a case report on a patient who had a successful reconstruction following an oncological resection. She had multiple resections and adjuvant treatment in the past which complicated the surgical planning. The uniqueness of this case lies not only in its prolonged clinical course but also the challenges met in a resource‐poor setting where chemotherapy and immunotherapy options are unavailable. Furthermore, the recurrent surgery and scarring complicate the clinical picture, propelling the surgeon to innovate and improvise.

Written informed consent was obtained from the patient for the publication of this case report and accompanying images.

## 2 Case Presentation

A 78‐year‐old lady first presented to the surgical oncology clinic of the Teaching Hospital Anuradhapura, Sri Lanka, in 2013 with a left‐side anterior chin lesion which was clinically malignant and proven on excision biopsy to be an infiltrating basal cell carcinoma with a deep resection margin of 0.5 mm and lateral resection margins involved by the tumor. She then underwent a lateral margin re‐excision (WLE), and a skin graft was performed. The re‐excision confirmed it to be infiltrating the deep and one radial margin involvement. This patient was then referred to the oncology clinic for adjuvant radiotherapy. 5000 Gy 20 cycles of radiotherapy was given with lead wax internal shielding to the jaw, teeth, and neck.

The patient later re‐presented to the surgical oncology clinic in December 2017 with a recurrent 1 cm lesion at the previous excision site. An excision biopsy was performed, yielding a histology of nodular basal cell carcinoma, 6 mm in size (pT1) with the tumor extending to the reticular dermis with 2 mm free resection margins. There was no perineural or lymphovascular invasion.

The patient had another recurrence in January 2021, and a wide local excision of the tumor was performed with primary closure. The histology revealed nodular infiltrative basal cell carcinoma, 6 mm in size with a depth of invasion of 4 mm and clear resection margins (deep < 0.1 mm, medial 0.1 mm, lateral 4 mm, inferior 2 mm) (pT1Nx). Following this, there was loss to follow‐up, and no adjuvant treatment was given.

**FIGURE 1:**
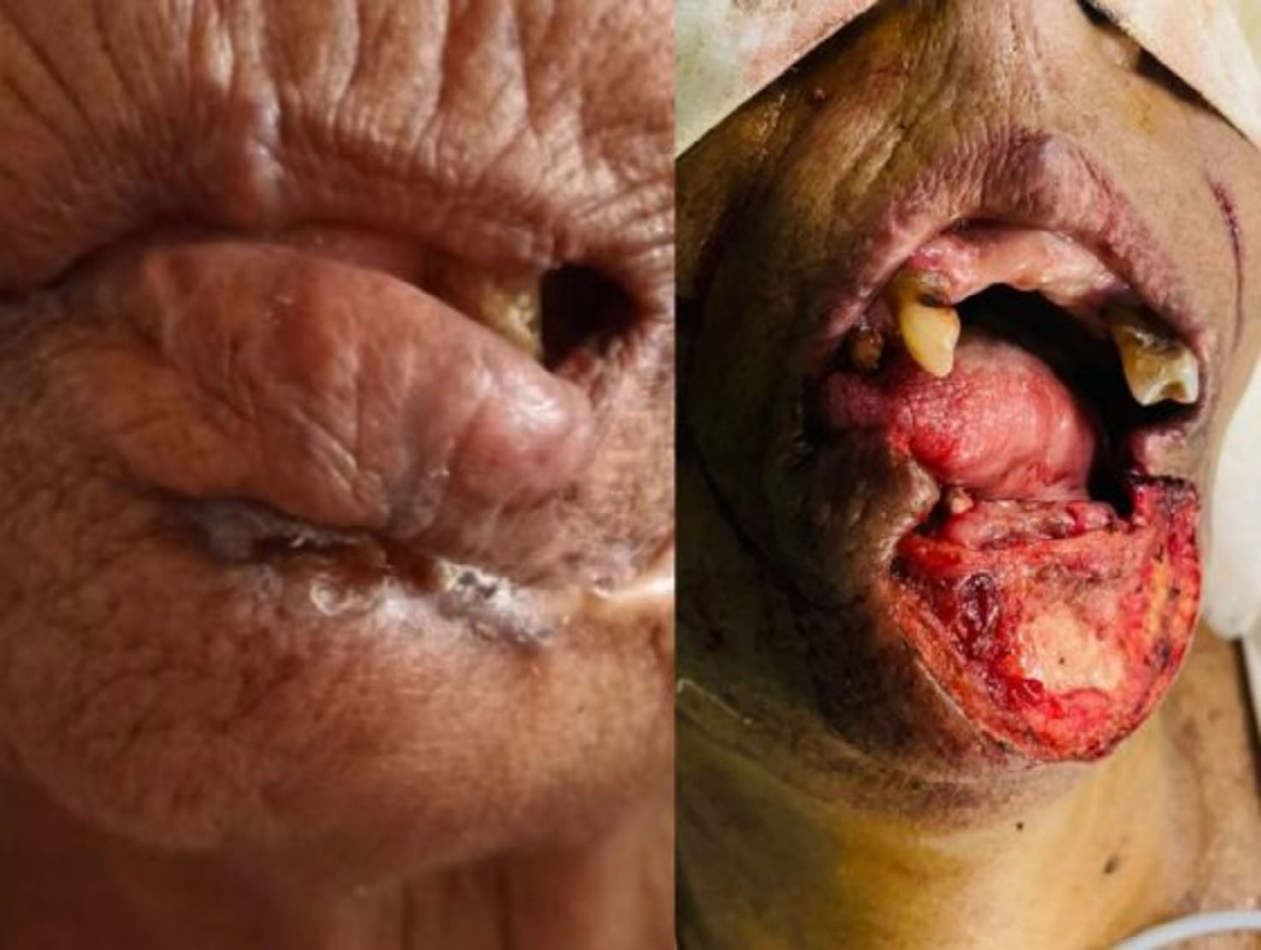
First shows the preoperative appearance and then the post resection appearance.

The author and the team first encountered the patient in 2023 with a similar lesion, on which a punch biopsy confirmed recurrence of adenoid basal cell carcinoma which was panCK, P40, CD10 positive, Bc12 weakly positive, and CK7 negative. An ultrasound scan of the neck did not show any suspicious lymphadenopathy. Due to previous surgeries, she had a scar on the lip as well as facial asymmetry and drooping of the lip on the left aspect with wrinkling. Figure 1 depicts the pre‐operative appearance of the patient, which culminated in mild incontinence and improper approximation of the lips due to the scar contracture over the pre‐existing scars. CT scan was also performed and confirmed no bony involvement of the region, but clinically it was determined to be lying in close proximity to the mandible.

The multi‐disciplinary meeting decision was to offer her surgical excision, but the challenge was to achieve adequate margins while maintaining continence and good functional as well as cosmetic outcome. It is worth noting that immunotherapy was not available as an option in the country at the time of presentation.

### 2.1 Surgical Technique

The surgical plan included performing a significant resection of the lower lip, which would invariably expose the mandible, and also harvesting the closest draining lymph nodes (Level I). Figure 1 shows the post‐resection appearance in which the mandible is exposed and there is over two‐thirds tissue loss of the lower lip. Dermoscopy and Moh micrographic surgical options are not available in Sri Lanka and the preoperative clinical marking was used to identify macroscopic resection margins (Resection margins of 1 cm from the gross palpable tumor).

The reconstruction aimed at recreating the lower lip in such a way that it maintained continence and covered the defect in the chin area. Over 70% of tissue loss of the lower lip was reconstructed using bilateral Karapandzic flaps with a risk of significant microstomia.

**FIGURE 2:**
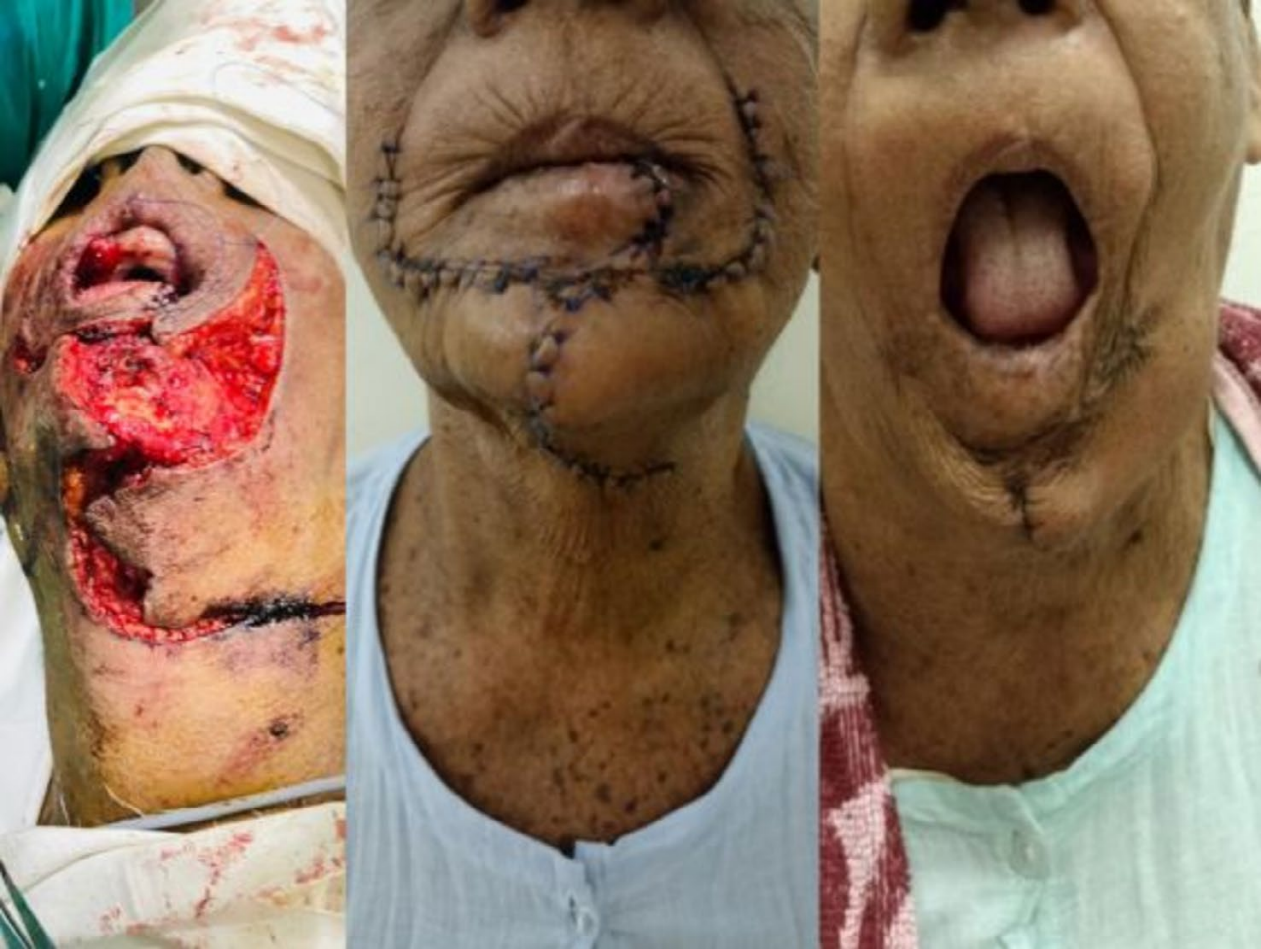
From left to right—appearance after raising the two flaps, appearance prior to discharge, 3 months post operative appearance.

For the second challenge of filling the chin defect, a bilobed random pattern rotational flap was marked in the neck and lymph node dissection was performed prior to raising it. Careful dissection was required at this point to ensure the integrity of the facial artery and its branches, especially the labial supply as this vessel plays a crucial role in supplying the combination of flaps. Figure 2 illustrates elevation of the bilobed flap from the neck which was transposed to the chin defect later and bilateral Karapandzic flaps are used to recreate the lower lip.

The patient was started on an anti‐sialogogue therapy for 5 days and fed via the NG for 3 days. Recovery was uneventful. Figure 2 depicts the appearance prior to discharge which was reassuring for good cosmesis as well as functionality. The dreaded microsomia was not profound and was satisfactory for the patient.

### 2.2 Outcome and Follow‐Up

Figure 2—is the appearance 3 months post‐operatively and she is back to her normal daily routine with no residual functional deficits and with an acceptable aesthetic outcome. Post‐operative histology confirms a basal cell carcinoma with squamous differentiation. The lesion was 1.5 cm with no lymphovascular or perineural invasion, satisfyingly with clear circumferential and deep margins (> 5 mm). Lymph nodes harvested were negative. The multidisciplinary decision was to not offer further treatment. The patient remains disease‐free at 18 months following surgery.

## 3 Discussion

Basal cell carcinoma (BCC) is the most common form of skin cancer, primarily affecting the head and neck region due to its association with ultraviolet (UV) light exposure. This case report discusses the challenges and complexities in managing recurrent and infiltrative BCC of the face, especially when multiple surgical interventions and adjuvant therapies have been utilized.

The patient's initial presentation in 2013 with an infiltrating BCC underscores the aggressive nature of this subtype, which often necessitates wide local excision (WLE) with adequate margins to prevent recurrence. While managing to achieve clear margins on multiple re‐excisions and adjuvant radiotherapies, the tumor recurred, reflecting the hardships of managing BCC with aggressive histological features. Furthermore, this highlights the importance of achieving the standard recommended margin for low‐risk BCC, which is 4–5 mm, while high‐risk cases requiring at least 6 mm margins.

Challenges faced: Recurrent cases, as portrayed by the patient's history, often involve compromised tissue integrity due to previous surgeries and radiation. Therefore, the subsequent surgical excisions are more challenging, as was evident in the 2023 surgery, where achieving adequate margins while maintaining functional and cosmetic outcomes posed significant difficulty. The devised plan, which involved extensive resection of the lower lip and reconstruction with bilateral Karapandzic flaps, is a testament to the need for meticulous surgical planning and technique. The Karapandzic flap is known for preserving the neurovascular supply and maintaining oral competence, hence, it was chosen to address the extensive tissue loss and ensure functional preservation [8].

Even though radiotherapy is not a recommended treatment option in the present day, the clinicians managing her in 2013 opted for radiotherapy as adjuvant modality possibly due to the unavailability of any other adjuvant treatment at the time. This approach, in our opinion, not only changed her disease‐free survival but also complicated future resections. Immunotherapy which is used widely and yields a better pathological response was not available in Sri Lanka during this period. In addition, most systemic chemotherapeutic agents were unavailable during this period of economic crisis and Covid 19 pandemic in the country. Therefore, surgical technique was seen as the best form of treatment if clear margins could have been achieved.

Review of other studies suggests that the recurrence of BCC after initial treatment is not uncommon. For instance, Bichakjian et al. [9] and Cameron et al. [1] reported that high‐risk BCC cases, especially those with infiltrative features, are prone to recurrence, requiring aggressive surgical approaches and advanced reconstruction techniques. However, compared to these reports, where chemotherapy and immunotherapy options were more readily available, this case underscores the challenges of managing such recurrences in settings where these treatments are not accessible.

Furthermore, Lear and Migden [10] emphasized the importance of a multidisciplinary approach to recurrent BCC management, involving dermatologists, oncologists, and plastic surgeons to optimize patient outcomes. This patient had a similar approach, despite the absence of certain systemic therapies. The follow‐up period and surgical precision showed in this case resulted in disease‐free survival of 18 months post‐surgery, which aligns with the findings of Patel et al. [11] on the effectiveness of advanced surgical interventions for high‐risk BCC.

Reconstruction Techniques: The bilobed random pattern rotational flap was used to cover the chin defect, which further demonstrates the necessity of versatile reconstructive options in complex facial surgeries. The combination of these reconstructive options ensured not only the closure of the defect but also the preservation of critical functions such as speech and swallowing. One of the challenges is to ensure that lingual branches are preserved during neck dissection and raising of the cervical flap to ensure the viability of the Karapandzic flaps. As alternative options, there wasn't a robust single pedicled or local flap that would have covered this defect but a radial forearm free flap would have been a viable option. However, the risk of flap necrosis in the background of radiotherapy was a concern. Furthermore, the superiority of karapandzic flaps in maintaining functionality and continence made it the preferred choice.

Histopathological Considerations: The post‐operative histology revealing basal cell carcinoma with squamous differentiation (metatypical BCC) reiterates the histological diversity within BCCs, which can affect prognosis and recurrence risk. Metatypical BCC, characterized by features of both BCC and squamous cell carcinoma (SCC), often exhibits more aggressive behavior and a higher risk for recurrence. The lack of lymphovascular and perineural invasion and the achievement of clear surgical margins (> 5 mm) in the final histopathology report provided a favorable prognosis for the patient, negating the need for further adjuvant therapy.

Long‐term Outcomes: The satisfactory recovery and disease‐free status 18 months post‐operatively underscore the importance of comprehensive surgical management and close follow‐up.

## 4 Conclusion

This case underscores the complexities involved in managing recurrent facial basal cell carcinoma (BCC), particularly in resource‐limited settings. The decade‐long recurrence pattern, along with functional and aesthetic challenges due to scar contracture, required advanced surgical and reconstructive techniques such as the Karapandzic and bilobed rotational flaps. Despite the absence of chemotherapy and immunotherapy options, the team achieved successful long‐term disease‐free survival and satisfactory cosmetic outcomes, demonstrating the efficacy of meticulous surgical interventions in complex BCC cases. This case highlights the surgical planning that ensured high‐quality care in low‐resource environments and in patients who are intolerant to systemic therapy or who opt for surgical resection alone. These findings suggest that even without access to the latest systemic therapies, functional and aesthetic success is possible with appropriate surgical expertise and long‐term follow‐up.

## Author Contributions

**T. H. Pathirana** (Lead Author, Surgeon): Primary author of the manuscript, led the clinical management of the patient and contributed significantly to writing the surgical technique and discussion sections. **V. Bandaranayake** (Co‐author, Surgeon): Assisted in surgical planning and reconstruction, contributed to the case report and reviewed the manuscript. **A. P. Nellihela** (Co‐author, Pathologist): Provided histopathological analysis and descriptions, contributed to the writing of the histopathological considerations section. **S. Nikeshala** (Co‐author, Oncologist): Involved in patient follow‐up and radiation treatment planning, contributed to the oncological aspect of the report. **T. Saranga** (Co‐author, Surgeon): Assisted in the reconstruction surgeries, provided surgical insights for the article, and contributed to the surgical technique section. **A. Abegunasekara** (Co‐author, Surgeon): Participated in the multidisciplinary team meetings and contributed to surgical management decisions. **S. Gunathilake** (Co‐author, Radiologist): Performed and interpreted imaging studies, contributed to the imaging and diagnosis sections. **N. J. Asanthi** (Co‐author, Pathologist): Contributed to histopathology review and finalizing the report, focused on the pathology section.

## Consent

Written informed consent for publication was obtained from the patient, and any identifying information has been anonymized in this report.

## Conflicts of Interest

The authors declare no conflicts of interest.

## Data Availability

The data that support the findings of this study are available from the corresponding author, T. H. Pathirana, upon reasonable request. Due to patient privacy and confidentiality agreements, data related to patient medical records and imaging are not publicly available.
